# Lesion segmentation in lung CT scans using unsupervised adversarial learning

**DOI:** 10.1007/s11517-022-02651-8

**Published:** 2022-09-20

**Authors:** Moiz Khan Sherwani, Aldo Marzullo, Elena De Momi, Francesco Calimeri

**Affiliations:** 1 Department of Mathematics and Computer Science, University of Calabria, Rende, Italy; 2 Department of Electronics, Information and Bioengineering (DEIB), Politecnico di Milano, Milan, Italy

**Keywords:** COVID 19, Unsupervised learning, Generative adversarial network, Image segmentation

## Abstract

Lesion segmentation in medical images is difficult yet crucial for proper diagnosis and treatment. Identifying lesions in medical images is costly and time-consuming and requires highly specialized knowledge. For this reason, supervised and semi-supervised learning techniques have been developed. Nevertheless, the lack of annotated data, which is common in medical imaging, is an issue; in this context, interesting approaches can use unsupervised learning to accurately distinguish between healthy tissues and lesions, training the network without using the annotations. In this work, an unsupervised learning technique is proposed to automatically segment coronavirus disease 2019 (COVID-19) lesions on 2D axial CT lung slices. The proposed approach uses the technique of image translation to generate healthy lung images based on the infected lung image without the need for lesion annotations. Attention masks are used to improve the quality of the segmentation further. Experiments showed the capability of the proposed approaches to segment the lesions, and it outperforms a range of unsupervised lesion detection approaches. The average reported results for the test dataset based on the metrics: Dice Score, Sensitivity, Specificity, Structure Measure, Enhanced-Alignment Measure, and Mean Absolute Error are 0.695, 0.694, 0.961, 0.791, 0.875, and 0.082 respectively. The achieved results are promising compared with the state-of-the-art and could constitute a valuable tool for future developments.

**Graphical abstract:**

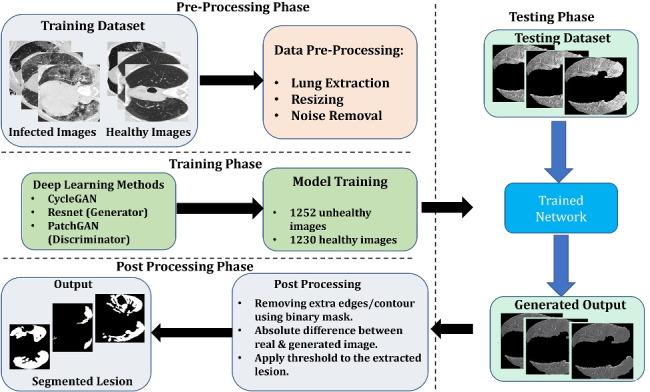

## Introduction

Due to the recent pandemic of new coronavirus disease (COVID-19), the world is experiencing a global health crisis [1, 2]. According to the Center for Systems Science and Engineering at Johns Hopkins University (updated 06-06-2022) 532,143,171 cases have been identified, including 6,299,644 deaths worldwide. For COVID-19 screening, accurate and fast segmentation of COVID-19 lesions within the lung, computed tomography (CT) images are widely used. However, the homogeneity of lesion tissues, the abnormality of lesion form, and similarities between the imaging features of lesions surrounding normal tissues make segmenting lung lesions in CT images difficult. Manually segmenting lesions is time-consuming and subjective due to variations in skills, knowledge, and experience across the operators [3–6].

In literature, several studies have been published that use machine learning (ML) (deep learning (DL)) techniques to detect COVID-19 infection regions within the lung region in CT images. COVID-19 recognition in CT slices is still challenging due to a few factors: (1) The significant diversity in texture, size, and location of the lesion in CT slices makes identification challenging; (2) gathering sufficient annotated material for training the DL model is problematic. There are a few studies in the literature that have examined unsupervised infection segmentation in CT slices, despite several methods to segment COVID-19 infection in clinical practice [7, 8]. Our group proposed an unsupervised approach for CT axial slices to overcome this issue. First, unhealthy (U) lung image slices are *translated* into healthy (H) image slices. Thus, the final segmentation result is the difference between translated and the original image (Table 1).

**Table 1:** Definition of abbreviation

Abbreviation	Definition
COVID-19	Coronavirus disease 2019
ML	Machine learning
DL	Deep learning
AI	Artificial intelligence
CT	Computed tomography
GAN	Generative adversarial network
G	Generator
*G*_*U*2*H*_	Generator (unhealthy to healthy)
*G*_*H*2*U*_	Generator (healthy to unhealthy)
D	Discriminator
*D*_*H*_	Discriminator for healthy domain
*D*_*U*_	Discriminator for unhealthy domain
U	Unhealthy data
H	Healthy data
GT	Ground truth

The remainder of the paper is structured as follows. After discussing the related work in Section 2, our group describes the material and proposed method in Section 3. Results are presented in Section 5 and discussed in Section 6. Section 7 draws our conclusions by summarizing the main results of the work.

## Related work

Generative adversarial networks (GANs)–based techniques [9] are applied on various domains like computer vision [10, 11], semantic segmentation [12], augmentation [13], image translation [14], and image synthesis.

In several cases, the segmentation of lesions in the medical images depends on texture information. The accuracy of the reconstruction is limited since the decoder looses some texture information [15]. Manjunath et al. [16] proposed the U-Net algorithm for automatic liver and tumor segmentation in CT images using supervised learning. The most recent work presented for the COVID-19 lesion segment using U-Net++ was proposed by Zhou et al. [17]. Most recent work focuses on image translation, segmentation [18], and generation of synthetic images [19] using supervised and semi-supervised learning to overcome this issue and to train the network for unsupervised segmentation, GAN, CycleGAN [14], and variational auto-encoder [20] are used. Other unsupervised approaches using similar generative models for image-to-image translation are DualGAN [21] and UNIT [22]. The GAN model can be used to segment several types of diseases, translate images from one image modality to another, and examine several other options.

Several COVID-19 segmentation techniques [23–26] based on artificial intelligence (AI) have recently been published and proved to be faster and more accurate and are given preference over the manual testing technique. At the beginning of the pandemic, Wang et al. [27] proposed a weekly supervised approach for COVID-19 classification. Wang et al. [28] took advantage of earlier consideration and extended their work for more discriminative COVID-19 detection. Ouyang et al. [29] proposed dual-sampling attention for COVID-19 diagnosis. CT imaging is a common and popular method for detecting and diagnosing lung disorders [30]. But it is harder for the COVID-19 segmentation task because of the absence of labeled data on the different textures of the infection [31].

Keshani et al. [32] detected the lung nodule in chest CT using a support vector machine (SVM) classifier. Wang et al. [33] segmented lung nodules from heterogeneous CT slices using a central focused convolutional neural network. In practice, crucial information can be obtained by segmenting different organs, and lesions from chest CT slices [34]. To overcome the issue of annotated data, Ma et al. [35] annotated 20 CT volumes from coronacases^1^ and radiopedia^2^. Fan et al. [36] proposed a semi-supervised architecture called Semi-Inf-Net. All these models relied upon information with annotations. Vidal et al. [37] provided the U-Net based transfer learning approach to diagnose the COVID infection in mobile devices. Saood et al. [38] utilized U-Net and SegNet to segment using CT scans. Yao et al. [39] introduced a method based on NormNet to differentiate between normal tissues and COVID-19-infected tissues. In this approach, NormNet was trained based on a fully unsupervised manner.

Ahrabi et al. [40] proposed the COVID-19 infection in lung CT using an unsupervised approach based on Auto-Encoder. Chen et al. [41] proposed the approach using unsupervised GAN for COVID-19 infection segmentation and domain adaptation. Another weekly supervised GAN-based approach for COVID-19 prediction on chest CT was proposed by Uemura et al. [42].

In this work, our group proposed an unsupervised approach using CycleGAN without any need for annotated data.

## Materials and methods

### Proposed architecture without attention guidance

Our task is to segment the infection out of the lung part, keeping the healthy part of the lung unchanged. The intuition is that if a COVID-19 infected lung image is correctly translated into its healthy-looking representation, the translator network has learned what a COVID lesion is and how it can be segmented.

Thus, the difference between the H and U images can be used to segment the lesion region within the image. Following the proposal of Zhu et al. [14], our main framework is based on CycleGAN as given in Fig. 2. Two generators (translators) and two discriminators make up the CycleGAN framework.

#### Generator (G)

G in our framework is the improved architecture of the ResNet architecture [43]. ResNets have exhibited significant performance across numerous benchmarks. ResNet contains seven residual blocks in our architecture, which provides identity mapping with information propagation bypassing the non-linear layer utilizing the shortcut connections. A residual block comprises convolutional, rectified linear unit (ReLU), and batch normalization layers. The detailed information about each layer with kernel, filters and stride is shown in Fig. 2 and Table 2. Our generators *G*_*U*2*H*_ and *G*_*H*2*U*_ consist of an encoder, transformer (residual block), and decoder. The residual block connects the encoder and decoder block. The features are under-sampled with the stride function in convolution layers and up-sampled in the de-convolution layers.

- Encoder: It extracts the feature from the images by using convolutional layers. The filter size plays an important role in this part because to extract the features of the input image, a window based on our filter size is moved considering the stride given for each step. Higher-level features of every image are extracted with a convolution layer.
- Transformer: It consists of two convolution layers with the ReLU activation function. This block ensures that the properties of the previous layer are not lost for the next layers. Otherwise, the output will not have the characteristics of the input image. Residual networks are used because it keeps the characteristics of the input size and shape of the object.
- Decoder: This step works like the inverse of the encoder part. It takes the feature vectors and converts them into low-level features. De-convolution or transpose convolution layers are used to achieve the required features. These low-level features are used in the final layer to generate the image.

**Table 2:** The network architecture of the ResNet generator

Layers	Input	Filter	Stride	Instance	Activation	Output
		size		normalization	function	
Convolution 1	256*256*1	7*7	1	X	ReLU	256*256*64
Convolution 2	256*256*64	3*3	2	X	ReLU	128*128*128
Convolution 3	128*128*128	3*3	2	X	ReLU	64*64*256
Residual Block(RB)x7						
Convolution 1 RB	64*64*256	3*3	1	X	ReLU	64*64*256
Convolution 2 RB	64*64*256	3*3	1	X	−	64*64*256
De-Convolution 1	64*64*256	3*3	2	X	ReLU	128*128*128
De-Convolution 2	128*128*128	3*3	2	X	ReLU	256*256*64
Convolution 4	256*256*64	7*7	1	−	tanh	256*256*1

#### Discriminator (D)

PatchGAN [44, 45] is used as D and the architecture is given in Fig. 2. The discriminators *D*_*U*_ and *D*_*H*_ consist of five convolutional layers that provide a single logit that tells if the image is H or U. Except for the first and last layers, all other layers are preceded by the batch normalization function. The ReLU activation function is utilized for all the hidden units. PatchGAN divides images into patches, and this method assigns a probability to the patches based on the content of the features rather than assigning a probability to each pixel. For this reason, its performance does not depend on the content but its features. The detailed information about each layer with kernel, filters, and stride is shown in Fig. 2 and Table 3. Its working is opposite of G, and due to that reason, sometimes the loss grows exponentially (discriminating all real images perfectly). It is advised to put some noise while training to train D much better.

**Table 3:** The network architecture of the PatchGAN discriminator

Layers	Input	Filter	Stride	Instance	Activation	Output
		size		normalization	function	
Convolution1	128*128*1	4*4	2	−	Leaky ReLU	128*128*64
Convolution2	128*128*64	4*4	2	X	Leaky ReLU	64*64*128
Convolution3	64*64*128	4*4	2	X	Leaky ReLU	32*32*256
Convolution4	32*32*256	4*4	1	X	Leaky ReLU	31*31*512
Convolution5	31*31*512	4*4	1	−	−	30*30*1

#### CycleGAN Network

The main neural network in our model is the G that takes an U image and generates a H image. The CycleGAN model has two mapping functions *G*_*U*2*H*_: $U \rightarrow H$ and *G*_*H*2*U*_: $H \rightarrow U$, as well as two associated adversarial discriminators *D*_*H*_ and *D*_*U*_. *G*_*U*2*H*_ is encouraged by *D*_*H*_ to translate U into outputs indistinguishable from domain H, while *G*_*H*2*U*_ is encouraged by *D*_*U*_ to translate H into outputs that are indistinguishable from domain U.

Cycle consistency loss is adopted in this network to regularize the mappings and transform the infected image into H by aiding the learning of the G. Generators learn and share additional information between U and H images using cycle consistency loss.

**Fig. 1:**
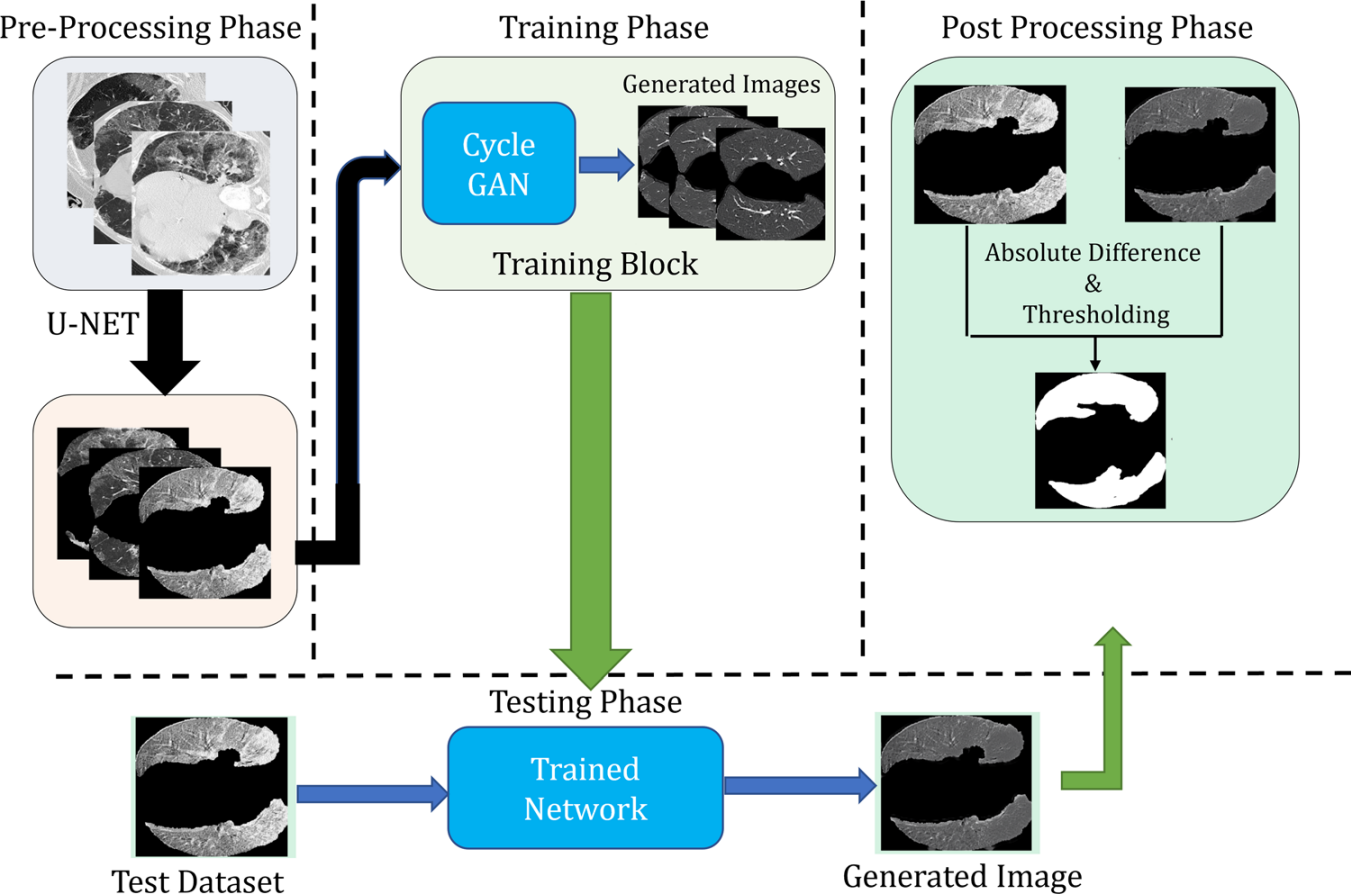
Proposed architecture is divided into three parts: Pre-Processing, Image Generation and Post Processing. Proposed CycleGAN for image generation phase is further described in Fig. 2

**Fig. 2:**
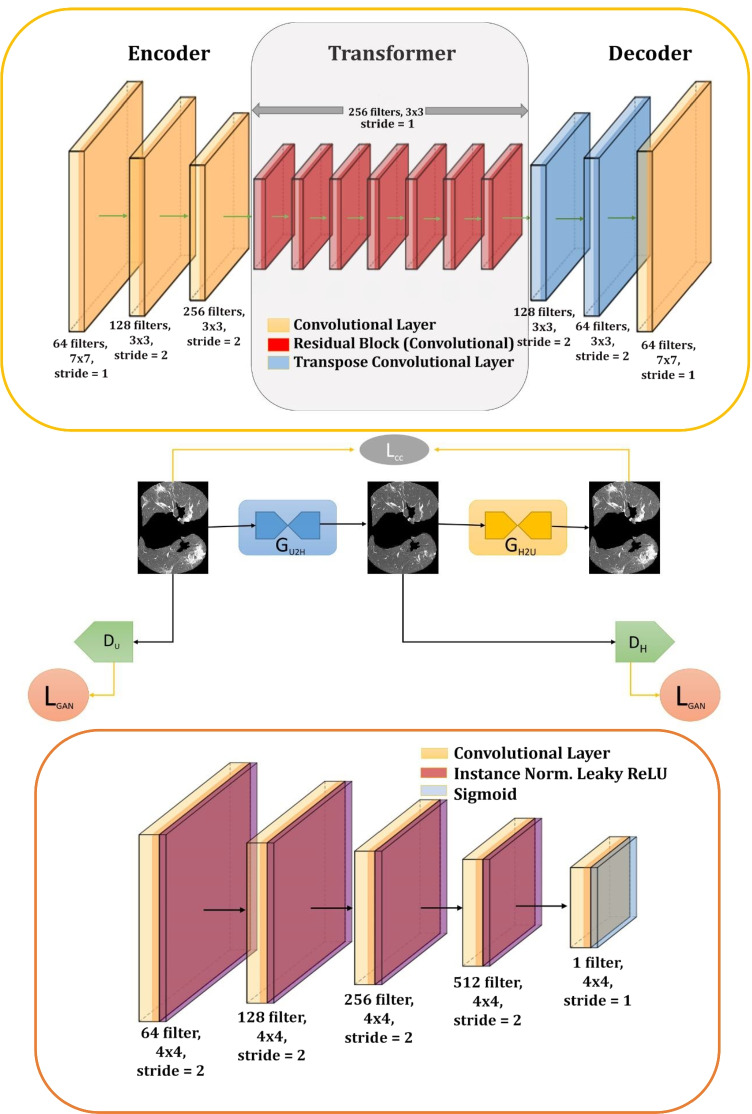
Proposed CycleGAN architecture. where *G*_*U*2*H*_ denotes Generator (unhealthy to healthy), *G*_*H*2*U*_ denotes Generator (healthy to unhealthy), *D*_*H*_ denotes Discriminator (healthy domain), *D*_*U*_ denotes Discriminator (unhealthy domain), *L*_*GAN*_ denotes Adversarial Loss, and *L*_*CC*_ denotes Cycle Consistency loss. Generator is described in the upper part of the image highlighted with yellow and Discriminator is described in lower part highlighted with pink
- **Post Processing phase**: This phase is designed to segment the final lesion based on the difference between the real image and generated image. As described in Fig. 1 The final segmentation is obtained with the following steps:
  - Remove the extra edges of the contours. The extra edge or contours are removed by using the binary mask of the input image.
  - Compute the difference between the real image and the generated image.
  - Apply threshold to the image received from the previous step to get the lesion part.

Our proposed architecture is given in Fig. 1. To further simplify, our group divided our proposed architecture into three phases:

- **Pre-processing phase**: In this phase, all images from the dataset are passed through the U-Net trained on the lung image dataset to extract only the lung part in the image.
- **Image Generation phase**: In this phase, the images are passed through the Image generation phase described in Fig. 1. These images are passed through our proposed CycleGAN described in Fig. 2

### Proposed architecture with unsupervised attention guidance

**Fig. 3:**
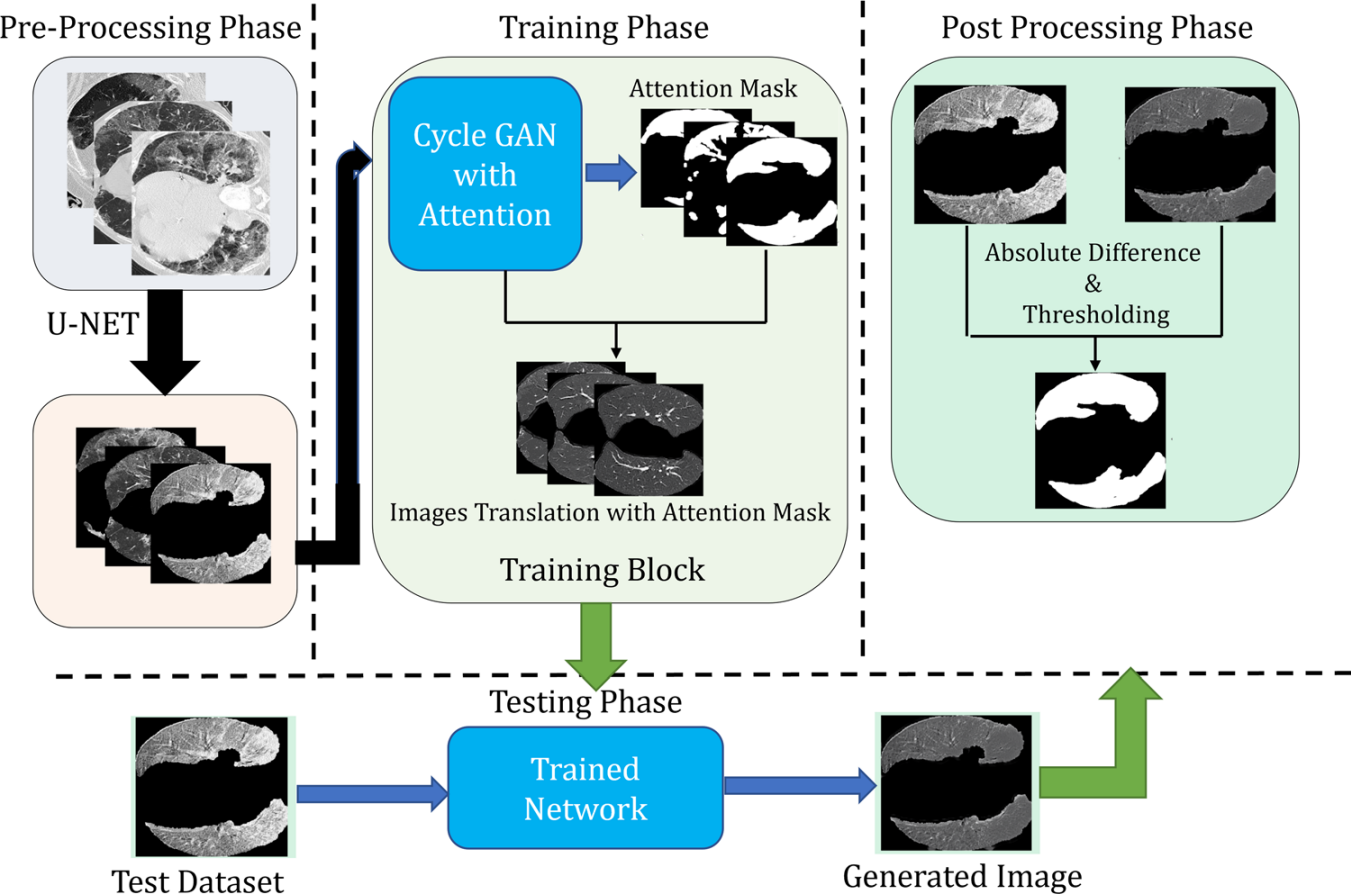
Proposed architecture with attention mask
- **Image translation using Mask** generates the image using the attention mask and it’s inverse as given in this equation:
  1 $$I = I_{a} \bigodot G_{U2H}(I) + (1-I_{a})\bigodot I$$
  where, I is the image, and *I*_*a*_ is the attention mask.
- **Post Processing phase** is similar to the above architecture without attention guidance.

This architecture is the extension of the architecture defined above. The following steps are followed to extract the lesion using this architecture:

- **Pre-Processing phase** is similar to the above architecture without attention guidance.
- **Attention Mask Generator phase** receives the image input shown in the Fig. 3 producing an attention map as proposed by Mejjati et al. [46]. Same G network is used for the unsupervised mask generation but in the last layer sigmoid function is used to generate the binary mask of only infected region avoid the healthy region of the lung.

This attention map provides us the pixel-wise information regarding the infection, and this provides the information to the network to focus on the only infected part of the lung CT. This attention network plays a key role because it locates the areas to be translated in each image, and the given area is translated significantly, keeping the healthy part unattended. Our approach would fail if the attention map provided all ones, which would cause the entire image to change. As long as the attention map shows all zeroes, the generated image would not change, and the G would never deceive the D.

#### Loss functions

Our main loss function consists of two different loss functions, which focus on minimizing over G and maximizing over discriminator. Our main loss function is given as:

2 $$L_{FULL} = L_{GAN} + \lambda_{CC}L_{CC}$$

where, *L*_*FULL*_ is the full loss, *L*_*GAN*_ is the adversarial loss, *λ*_*cc*_ is constant parameter used to weight the forward and backward cycle loss and *L*_*cc*_ is the cycle consistency loss. Explanation of each terms are given below:

#### Adversarial loss (*L*_*GAN*_)

Our group implemented adversarial losses to both mapping functions. For the mapping function G: U to H and its D *D*_*H*_, the loss function is given as:

3 $$\begin{array}{@{}rcl@{}} L_{GAN}(G_{U2H}, D_{H} , U, H ) &=& \mathbb{E}_{H\overset{\sim}p_{data}(H)} [log D_{H} (H)] + \\ &&\mathbb{E}_{U\stackrel{}{\sim}p_{data}(U)} [log(1 - D_{H} (G(U))] \end{array}$$

where, *G*_*U*2*H*_ attempts to generate U images that appear to be like images from H images , while Discriminator (*D*_*H*_) expects to recognize generated samples *G*_*U*2*H*_(*U*) and real sample H. G aims to limit this goal against the D that attempts to maximize it, i.e., $min_{G_{U2H}}$ $max_{D_{H}}$ *L*_*GAN*_ (*G*_*U*2*H*_, *D*_*H*_, U, H). A similar adversarial loss for the mapping function *G*_*H*2*U*_: $H \rightarrow U$ and its discriminator *D*_*U*_ also: i.e., $min_{G_{H2U}}$ $max_{D_{U}}$ *L*_*GAN*_ (*G*_*H*2*U*_, *D*_*U*_, H, U).

#### Cycle consistency loss (*L*_*cc*_)

Our group adopted a cycle consistency term [35] to generate H images from infected images and aid learning of *G*_*H*2*U*_ and *G*_*U*2*H*_. Adversarial loss alone can not guarantee that the learned function can map one U into a H. The cycle consistency loss function allows us to communicate more information between H and U images. The bidirectional cycle consistency learns a better model than unidirectional consistency terms alone. Our group needed to implement the instinct that these mappings ought to be inverts of one another and that the two mappings ought to be bijections. Cycle consistency loss encourages *G*_*H*2*U*_(*G*_*U*2*H*_(*U*)) ≈ *U* and *G*_*U*2*H*_(*G*_*H*2*U*_(*H*)) ≈ *H*. Our main loss function consists of two different loss functions, which focus on minimizing over G and maximizing over discriminator.

## Experimental analysis

### Dataset description

Two medical imaging datasets were used for the evaluation of lung lesion segmentation in axial CT scans. Other recent open COVID-19 CT Dataset with automatic classification of lung tissues for radiomics is also made available by Zaffino et al. [47].

#### A. COVID-19 CT Segmentation dataset

3, which consists of 100 axial CT images from different COVID-19 patients. All CT images were collected by the Italian Society of Medical and Interventional Radiology^4^. The radiologist segmented all CT images using the labels for lung infection detection. However, because it is the first open-access COVID-19 dataset, it has a limited sample size of only 100 CT images. This dataset was used for the baseline methods, and our group tested our approach with other baseline methods using this dataset as the test set.

#### B. SARS-COV-2 CT scan dataset

5, which consist of 1252 CT scans that are positive for COVID-19 and 1230 CT scans for patients not infected by COVID-19 scans. This data has been collected from real patients in a hospital in Sao Paulo, Brazil. This dataset is the unpaired dataset for COVID-19 U and H images. Our group used this dataset to train our network.

**Fig. 4:**
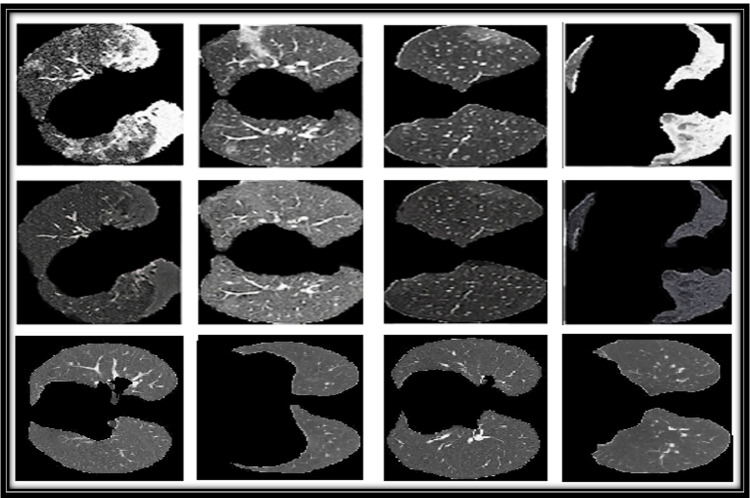
First row contains COVID-19 infected images, while second row contains there respective healthy translation and last row contains the healthy images used for training

As seen in Fig. 4, the COVID-infected areas on CT images are of various shapes and sizes. Furthermore, many CT images are collected so that more noise-like artifacts are introduced. As a training dataset, 1275 U lung CT images were used, as well as 1230 H lung CT images, while as a testing dataset, all images from the COVID-19 dataset were used.

#### Baselines

The proposed method was compared with INF-NET [36], Semi-INF-Net [36] and other classical segmentation models commonly used for segmentation in the medical domain. i.e., U-Net [48], U-Net++ [18].

#### Evaluation metrics

Our group evaluated the results using some widely adopted metrics, i.e., Dice Similarity Coefficient (DSC), Sensitivity (Sen.), Specificity (Spec.), Precision (Prec.), Structure Measure, Enhance-alignment Measure, and mean absolute error (MAE).

1. **Dice Similarity Coefficient.** This metric is widely used to assess the repeatability of manual segmentations as well as the accuracy of automated probabilistic fractional segmentation in terms of spatial overlap.
  4 $$DSC= \frac{2TP}{2TP + FP + FN}$$
2. **Sensitivity.** The metric that measures a model’s ability to predict true positives in each accessible category is called sensitivity.
  5 $$Sensitivity = \frac{TP}{TP + FN}$$
3. **Specificity.** The metric that measures a model’s ability to predict true negatives in each accessible category is called specificity.
  6 $$Specificity= \frac{TN}{TN + FP}$$
  where, TP= algorithm correctly classified the pixel comparable to ground truth (GT). FP= pixels not classified as lung in GT, but classified as lung by algorithm. TN= pixels not classified as lung in GT and by algorithm. FN= pixels classified as lung in GT, but not classified as lung by algorithm.
4. **Structure Measure** (***S***_***α***_). Fan et al. [49] provided the metric to evaluate region-aware and object aware structural similarities between the generated image and the GT. This metric offers salient object detection evaluation. Our group reported *S*_*α*_ using the default settings suggested in the paper.
  7 $$S_{\alpha}=(1-\alpha)*S_{0}(S_{p},G)+\alpha*S_{r}(S_{p},G)$$
  where, *S*_0_ = Object aware similarity. *S*_*r*_ = Region aware similarity. *α* is a balance factor between *S*_0_ and *S*_*r*_. *S*_*α*_ using the default setting (where *α* = 0.5) as suggested in the original paper. [49]
5. **Enhance-Alignment Measure** (***E***_***ϕ***_). Fan et al. [50] recently proposed this metric to evaluate both local and global similarities between two binary map. The formula is provided below:
  8 $$E_{\phi}=1/w\times h{\sum\limits_{x}^{a}}{\sum\limits_{y}^{h}}\phi(S_{p}(x,y),G(x,y))$$
  where, pixel coordinates in GT are given as (*x*, *y*), and width and height as w and h. Enhanced alignment matrix is mentioned as symbol *ϕ*. A binary mask using threshold is converted by prediction Sp to obtain a set of *E*_*ϕ*_.
6. **Mean Absolute Error (MAE).** This metric is used to determine the image’s pixel-by-pixel inaccuracy between the image and GT, which is defined as:
  9 $$MAE=1/w\times h{\sum\limits_{x}^{a}}{\sum\limits_{y}^{h}}|S_{p}(x,y)-G(x,y)|$$

### Training phase

During the training phase, only one input channel was used. All images were set to grayscale as we used our framework with the grayscale parameter enabled.

The described workstation was used to run the following tests: Intel(R) Core(TM) i7-5930K CPU @ 3.50GHz , Ubuntu 16.04.6 LTS, CUDA tools, release 11.0, V11.0.194, NVIDIA Quadro p6000 24gb. For training, our group set *λ* cc = 10, to optimize, our group used ADAM optimizer with *β*_1_ = 0.5 and *β*_2_ = 0.999. The learning rate to train the network for 200 epochs is fixed at 2 × 10^− 4^ and batch size = 8. The implementation of the code is based on pytorch, and it works well with torch version 1.4, torchvision version 0.5, dominate version 2.4.0, and visdom version 0.1.8.8. The implementation and dataset of the code are available on https://github.com/mksherwani/Unsupervised-attention-based-CycleGAN-for-COVID-lesion-segmentation.git

## Results

Our group compared the experimental results of our approach with some of the well-known previous baseline approaches, including U-NET, U-NET++, INF-Net, and Semi INF-Net. The quantitative results based on the defined metrics are given in Table 4. Table 4 depicts the best results in favor of our approach concerning the unsupervised learning keeping in mind the ground truth images.

**Table 4:**
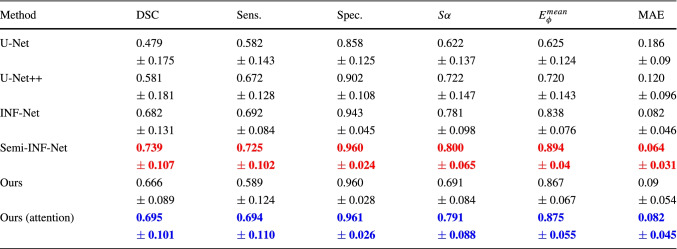
Quantitative results of infected region on our COVID-19 data

For each column, the  score is highlighted in red color and  best values are highlighted in blue color

**Fig. 5:**
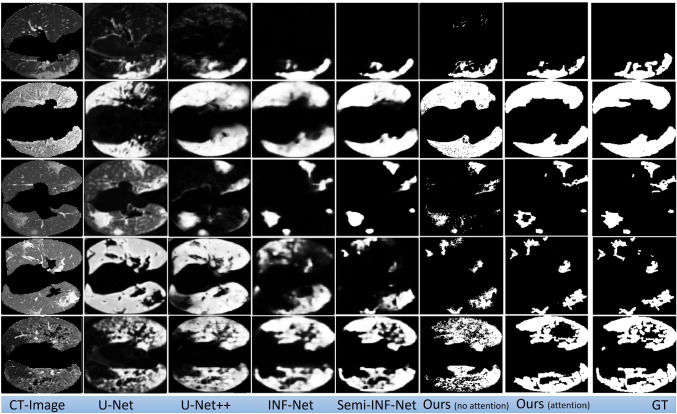
Visual comparison of lung lesion segmentation result

The results described in Table 4 and Figure 5 are the best results achieved by training our model. In order to obtain this result, several trainings are run to determine the best optimized model. During hyper-parameter tuning, the loss values of the generators and discriminators were observed along with the synthetic images generated during training. Some results from the models while hyper-parameter tuning is shown in Table 5

**Table 5:** Quantitative results of infected region on our COVID-19 data based on different training for hyper-parameter tuning

# of trainings	DSC	Sens.	Spec.	*Sα*	$E_{\phi }^{mean}$	MAE
1	0.672	0.646	0.958	0.747	0.869	0.085
	± 0.125	± 0.111	± 0.028	± 0.082	± 0.064	± 0.049
2	0.683	0.65	0.958	0.763	0.87	0.088
	± 0.133	± 0.091	± 0.047	± 0.081	± 0.071	± 0.049
3	0.668	0.593	0.956	0.703	0.869	0.088
	± 0.112	± 0.09	± 0.029	± 0.094	± 0.059	± 0.05
4	0.685	0.691	0.960	0.783	0.844	0.083
	± 0.129	± 0.117	± 0.038	± 0.087	± 0.063	± 0.047

As given in the Table 5, results from some of the optimized models using different configuration for hyper-parameter tuning is given. Parameters for training phase are given in the section based on training phase. But based on the table, we made changes in some parameters while training 1, 2, 3, and 4.

For train 1, we used the Stochastic Gradient Descent (SGD) as optimizer replacing ADAM with same parameters. For train 2, we changed the learning rate = 0.00005 and also the batch size = 16. For train 3, we split the train and test set. For training 75% of the images were used to train the network and 50% for testing. For train 4, we used ADAM optimizer changing its *β*_1_ and *β*_2_ also changing the batch size = 4 and epoch = 120.

### Ablation study

Ablation studies depend on removing certain parts of the network to understand the impact and the behavior of the network [51]. The thought is that certain boundaries of a referenced network contribute very little or, on the other hand, nothing to the network’s performance, making them irrelevant and, in this manner, ready to be taken out. Our group needs to utilize this ablation approach not to work on the size and speed of a neural network but to get capabilities into the effect of each progression on the performance, resulting in an interpretable model. Table 6 shows the Dice Similarity Coefficient scores in the ablation study.

**Table 6:** Quantitative results based on different ablation studies

Method	DSC	Sens.	Spec.	*Sα*	$E_{\phi }^{mean}$	MAE
Impact of						
Generator model	0.666	0.685	0.921	0.756	0.786	0.097
Impact of						
Loss function	0.608	0.677	0.911	0.732	0.760	0.102
Impact of						
Hidden network layers	0.672	0.689	0.858	0.788	0.821	0.099
Ours	0.695	0.694	0.961	0.791	0.875	0.082

#### Impact of Generator model

Several studies have been conducted to compare different G models. Yu et al. [52] presented the study for image synthesis comparing different G models, including U-Net, ResNet, and ResU-Net. Another study conducted by Lee et al. [53] provided the optimal generative model for the CycleGAN architecture for medical image synthesis. In the study, ResNet performed better in terms of image quality while U-Net quality was not comparable to ResNet, and it took a much longer time to train on the same sample and consumed high memory. Their study also concluded that U-Net performed much better for image segmentation than ResNet. Based on the intuition and the literature review, our group also tested U-Net as G to compare the difference between the generated images and the network’s performance with our proposed ResNet. As per the results given in Table 6, our proposed approach performance in terms of all the metrics performed better than U-Net but the training time with U-Net was much faster as compared to ResNet. Based on the results produced with both the Gs, our group used ResNet for our training and testing because the results proved to be better than U-Net.

#### Impact of Loss function

In this study, our group introduced a loss function to avoid using the attention mask G. The main idea of using that loss function was to test if the image generation’s performance is improved compared to our proposed approach. For this study, our group used images with annotations. The loss function was based on Binary Cross-Entropy (BCE), which compared the infected part with annotation while training the network. For each image fed while training, the generated image was subtracted from the real image, and the remaining part was compared with the ground truth. The loss function was given as follows:

10 $$Loss = BCE(X , C) * weight$$

here X = real image - generated image and C = ground truth of the image fed. A constant value is input as weight to avoid the error of zero (if the segment looks exactly the same as the ground truth).

#### Impact of Hidden network layers

ResNet with different number of residual blocks can have impact on the network. Changing the residual block can improve or affect the accuracy of the image translated. In this study, our group studied the impact of changing the residual block and its layer on the image generation process as proposed by Wu et al. [54], and Yao et al. [55]. Our group trained the ResNet network with the different number of hidden layers in the residual block to improve the performance in terms of improved healthy representation and improve our experiment’s training time. Several studies have been conducted based on the different layers in the residual block. Some studies were also conducted based on the convolutional layer in the residual block, but the results show that our approach resulted in higher DSC and other evaluated metrics.

## Discussion

CT scans for detecting any disease, including COVID-19, is easier because it is available at any nearby hospital. Our GAN-based approaches provided promising performance. Considering the lack of freely available annotated data for training a neural network, this approach could be beneficial because it does not require annotated data for diagnosing any disease. Some of the H samples generated by both the approaches used are given in Fig. 4.

Our group compared the lesion segmentation with baseline methods U-Net, U-Net++, INF-Net, and Semi-INF-Net. Qualitative results based on baseline methods are shown in Fig. 5. The qualitative result shows that U-Net and U-Net++ segmentations were blurry, and most of the lesion segments are missing. However, comparing INF-Net and Semi-INF-Net lesion segmentation was better than U-Net and U-Net++, but Semi-INF-Net outperformed all other methods. Comparing our approaches with Semi-INF-Net, the lesion segment is comparable to the ground truth.

More detailed quantitative results are given in Table 4 based on different evaluation metrics. It shows that our methods outperformed all baseline methods except Semi-INF-Net, and it has extracted the infection region closely comparable to the GT with fewer mis-segmented tissues. Even though quantitative results show that our approach did not improve the results compared to Semi-INF-Net, our approach shows that it can train the network without the need for annotation, and the network can be trained with the unpaired data. This approach can be utilized for different medical image infection segmentation.

With other approaches used as the baseline, these results are not possible without the paired data or annotation, and the baseline methods showed the limitations that make our approach favorable. U-Net has provided unsatisfactory results with a large number of mis-segmented tissues. However, U-Net++ performed better than U-Net. Comparing INF-Net and Semi-INF-Net, Semi-INF-Net performance is much better than INF-Net. Our approach with unsupervised attention CycleGAN performed better than the CycleGAN without attention.

After conducting the ablation study based on the result evaluated on the testing dataset, our group observed that the proposed approach had the highest score for all evaluated metrics compared with the ablation studies conducted. Our proposed approach result showed the effectiveness of the model.

### Limitations

**Fig. 6:**
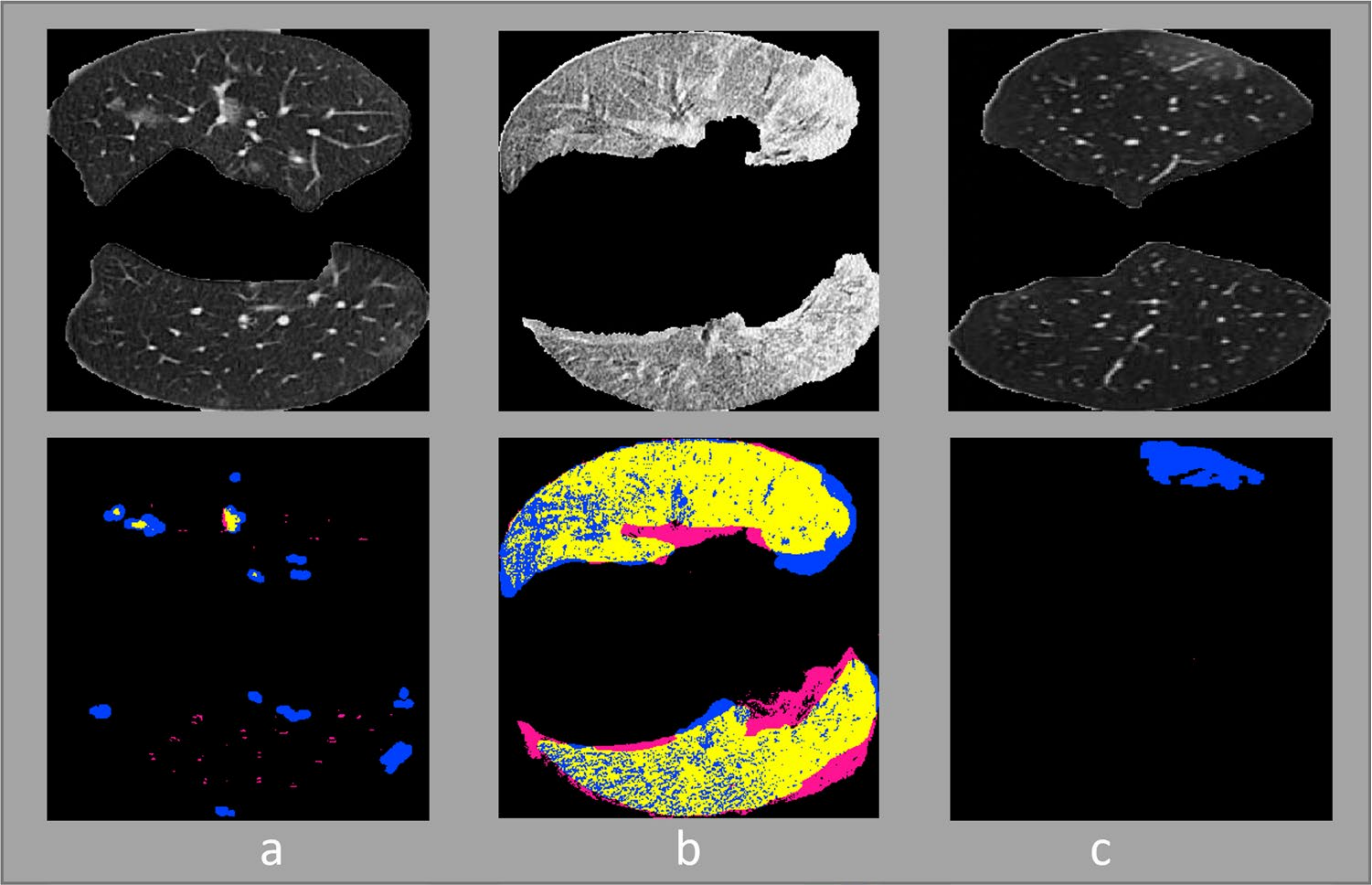
Failure cases in the segmentation. where, upper row contains testing images and lower row contains infection segmentation overlaid on the ground truth. (Pixels representing true positive = yellow, false positive = pink, false negative = blue, and true negative = black.)
- The network misidentified some of the pixels in the infected part considering it to be healthy tissues as shown in Fig. 6 (image b).
- Since the hyper-parameter tuning was performed manually, our group consider this point as a limitation and this limitation should be considered in the future prospects. Automatic hyper-parameter tuning is one of the important aspect because it help us optimize the hyper-parameters and without it, we can have sub-optimal results.

Despite the improvement contrasted with existing unsupervised lesion segmentation techniques, there is still an open problem between our approach and other supervised and unsupervised techniques. After studying some failure segmentation, our group concluded:

- The network missed some small infections as given in Fig. 6 (image a & c) and consider the infected region as healthy tissues.

## Conclusion

Our paper proposed a GAN-based approach to translating the lung CT containing COVID lesion into the equivalent healthy representation of that lung image, which utilizes the generators and discriminators for the image translation. Moreover, our paper also extended this solution, using an unsupervised attention mask generator, which uses the same network as above but also generates the attention masks of only the lesion region to improve the translation without changing the healthy part of the lung in CT. The proposed models can recognize the pixels with infection and healthy tissues.

To improve the performance of COVID-19 lesion segmentation, our future approach would be to extend this work in the following aspects: (1) To improve the loss function for D that can improve the discrimination process and better images can be generated. (2) To extend this approach to train using semi-supervised learning. Besides COVID-19 data, it is also possible to use this approach with other medical image datasets. This approach can generate a healthy representation of any lesion/tumor region. Our group will investigate other medical data in the future as well.

## Footnotes

1 https://coronacases.org/

2 https://radiopaedia.org/articles/

3 http://medicalsegmentation.com/covid19/

4 https://www.sirm.org/category/senza-categoria/covid-19

5 www.kaggle.com/plameneduardo/sarscov2-ctscan-dataset
